# Integrative morphological and DNA barcoding analysis of *Forcipomyia* (*Lasiohelea*) midges: cryptic diversity and first molecular detection of multiple *Leishmania* species in a leishmaniasis-endemic focus in Southern Thailand

**DOI:** 10.1186/s13071-026-07544-5

**Published:** 2026-06-30

**Authors:** Mawra Nadeem, Sakone Sunantaraporn, Chulaluk Promrangsee, Chatchapon Sricharoensuk, Rinnara Ampol, Rungfar Boonserm, Puckavadee Somwang, Pathamet Khositharattanakool, Padet Siriyasatien, Kanok Preativatanyou

**Affiliations:** 1 International Program of Medical Sciences, Faculty of Medicine, Chulalongkorn University, Bangkok, 10330 Thailand; 2 Center of Excellence in Vector Biology and Vector-Borne Disease, Chulalongkorn University, Bangkok, 10330 Thailand; 3 Department of Parasitology, Faculty of Medicine, Chulalongkorn University, Bangkok, 10330 Thailand; 4 School of Medicine, Mae Fah Luang University, Chiang Rai, 57100 Thailand; 5 Biomedical Technology Research Group for Vulnerable Populations, Mae Fah Luang University, Chiang Rai, 57100 Thailand

**Keywords:** Biting midges, *Forcipomyia*, *Lasiohelea*, DNA barcoding, Phylogeny, Species delimitation, Nanopore metabarcoding, *Leishmania*

## Abstract

**Background:**

Ceratopogonid midges of the genus *Forcipomyia* (subgenus *Lasiohelea*) are small hematophagous insects widely distributed across tropical regions. In Australia, developmental stages of *Leishmania* (*Mundinia*) *macropodum* have been observed in *Forcipomyia* (*Lasiohelea*), suggesting that biting midges may play a role in *Leishmania* transmission beyond traditional sand fly vectors. However, in Southeast Asia—where leishmaniasis caused by *Mundinia* species is an emerging autochthonous disease in humans—fundamental information on *Forcipomyia* (*Lasiohelea*) diversity and its association with *Leishmania* remains limited.

**Methods:**

An integrated morphological-molecular approach was employed to characterize *Forcipomyia* (*Lasiohelea*) midges collected using sweep nets from a leishmaniasis-endemic area in Nakhon Si Thammarat Province, Southern Thailand, near the residence of a patient with locally acquired cutaneous leishmaniasis, during January and June 2025. Morphological examination of mandibular dentition, sensory pits, cibarial armature, and spermathecal structure was combined with mitochondrial *cox*1 barcoding, Bayesian and maximum likelihood phylogenetic analyses, and species delimitation methods (ASAP and mPTP). Because females of the subgenus *Lasiohelea* possess a single spermatheca, only single-spermatheca females were selected for species identification. Specimens identified as *Lasiohelea* were subsequently screened for *Leishmania* using 18S rRNA-qPCR and ITS1-PCR, followed by nanopore-based ITS1 metabarcoding for species-level identification. Vertebrate blood meal sources were also characterized using vertebrate *cox*1 metabarcoding.

**Results:**

From 264 collected midges, 72 female specimens with a single spermatheca were selected for analysis. Integrated morphological and molecular data identified seven *Lasiohelea* specimens forming four genetic clusters, comprising *F.* (*L.*) *parvitas* (*n* = 2) and three lineages closely related to *F.* (*L.*) *peditata* (*n* = 2), *F.* (*L.*) *humilavolita* (*n* = 2), and *F.* (*L.*) *taiwana* (*n* = 1). These were clearly separated from non-*Lasiohelea* taxa, including *F.* (*Euprojoannisia*) *fuscimana* and two unclassified Ceratopogonidae lineages. Phylogenetic and species delimitation analyses revealed cryptic genetic diversity despite morphological similarity. *Leishmania* DNA was detected in six of seven *Lasiohelea* specimens. Nanopore ITS1 metabarcoding identified autochthonous species (*L.* (*Mundinia*) *martiniquensis* and *L.* (*M.*) *orientalis*) and additional *Leishmania* species (*L.* (*Leishmania*) *amazonensis* and *L.* (*L.*) *major*), including mixed-species detections in four specimens. A single engorged specimen contained DNA from red junglefowl (*Gallus gallus spadiceus*).

**Conclusions:**

This study provides the first integrated characterization of *Forcipomyia* (*Lasiohelea*) diversity and associated *Leishmania* detection in Southeast Asia. The results identified several putatively distinct species within *Lasiohelea* and provide evidence of natural exposure of these midges to multiple *Leishmania* species, suggesting a complex parasite-midge association and parasite co-circulation in the local environment. Although vector competence was not assessed, these findings suggest that *Forcipomyia* (*Lasiohelea*) may be involved in the circulation of *Leishmania* parasites and may represent promising candidates for further investigation of vector competence. Future studies focusing on host associations, parasite development, and experimental transmission are needed to clarify their epidemiological significance.

**Graphical Abstract:**

**Figure:**
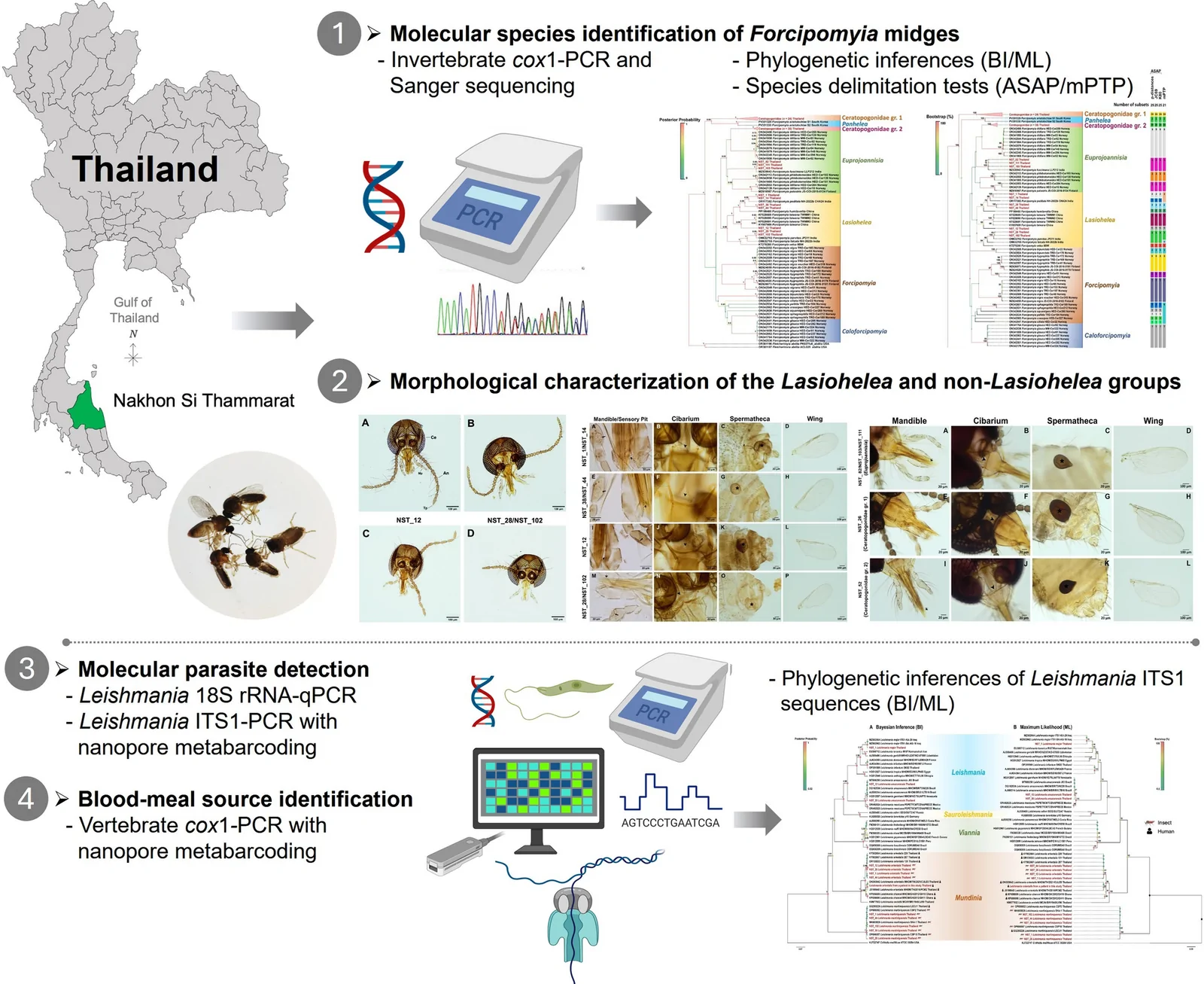

**Supplementary Information:**

The online version contains supplementary material available at https://doi.org/10.1186/s13071-026-07544-5.

## Background

Several members of the family Ceratopogonidae (order Diptera), including species of the genera *Austroconops*, *Culicoides*, *Leptoconops*, and *Forcipomyia* (*Lasiohelea*), are hematophagous insects known to feed on a wide range of vertebrate hosts [1]. Among these, biting midges of the genus *Culicoides* are of medical and veterinary importance, as they are recognized biological vectors of numerous pathogens, including arboviruses, bacteria, protozoa, and filarial nematodes, that affect humans, livestock, and wildlife worldwide [2–4].

There has been a notable increase in reported cases of leishmaniasis in Southeast Asia, with Thailand recognized as a major hotspot [5–8]. The indigenous *Leishmania* species in Thailand belong to the newly described subgenus *Mundinia* [9]. Besides the traditional phlebotomine sand flies, *Leishmania* (*Mundinia*) *martiniquensis* and *Leishmania* (*Mundinia*) *orientalis* have been molecularly detected in various *Culicoides* species collected from autochthonous leishmaniasis foci in Northern and Southern Thailand [10–16]. Previous experimental infections have shown that multiple *Leishmania* (*Mundinia*) species can complete their developmental cycle, including the production of infective metacyclic promastigotes, in the midgut of *Culicoides sonorensis* under laboratory conditions [17–19]. These findings suggest that *Culicoides* midges may act as competent or alternative vectors in the transmission of autochthonous leishmaniasis in Thailand.

In addition to *Culicoides*, other ceratopogonid genera, such as *Forcipomyia* (*Lasiohelea*) have also been implicated in the transmission of *Leishmania* (*Mundinia*) parasites. In Australia, *Leishmania* (*Mundinia*) *macropodum*, a marsupial-infecting parasite, was molecularly detected in up to 15% of field-collected *Forcipomyia* (*Lasiohelea*) specimens [20, 21]. Microscopic examinations have also confirmed the presence of metacyclic promastigotes colonizing the stomodeal valve of these diurnal midges, demonstrating their ability to support the parasite’s complete developmental cycle [20]. These findings support the role of *Forcipomyia* (*Lasiohelea*) midges as potential vectors of cutaneous leishmaniasis in red kangaroos (*Macropus rufus*) in Australia [20, 21]. Consequently, it is plausible that *Forcipomyia* (*Lasiohelea*) species elsewhere could also act as vectors for other *Leishmania* (*Mundinia*) species.

Despite emerging evidence supporting the role of *Culicoides* midges in leishmaniasis transmission, information on the diversity, blood-feeding behavior, and vector competence of *Forcipomyia* (*Lasiohelea*) outside Australia remains limited. In Southeast Asia, more than 168 *Culicoides* species have been described, including more than 100 in Thailand [22]. However, there are no confirmed reports documenting the presence of hematophagous *Forcipomyia* (*Lasiohelea*) species in the country. Consequently, three major knowledge gaps remain. First, the species diversity and taxonomic composition of *Forcipomyia* (*Lasiohelea*) in Thailand are poorly understood due to the lack of integrated morphological and molecular investigations. Second, the hematophagous behavior and vertebrate host associations of these midges remain largely undocumented, limiting our understanding of their potential contact with reservoir hosts and humans. Third, their vector competence and possible role in the transmission of *Leishmania* (*Mundinia*) parasites have not been investigated. Studies from nearby regions, such as Taiwan, have demonstrated that *Forcipomyia* (*Lasiohelea*) species, including the human-feeding midge *F.* (*L.*) *taiwana*, exhibit behaviors and habitat preferences potentially relevant to disease transmission [23, 24], though their definitive role as vectors in Southeast Asia requires further research.

Therefore, the present study aimed to address these knowledge gaps by employing an integrative morphological and molecular approach to characterize the diversity of *Forcipomyia* (*Lasiohelea*) midges collected near the residence of a cutaneous leishmaniasis patient in Nakhon Si Thammarat Province, Southern Thailand. The study site was selected due to a locally acquired case of cutaneous leishmaniasis, providing an epidemiologically relevant setting for investigating potential vector species, host-feeding patterns, and *Leishmania* circulation in the local environment. Molecular screening was conducted to detect *Leishmania* in collected specimens, and blood meal analysis was performed to identify vertebrate host species, thereby providing insights into possible ecological links among midges, vertebrate hosts, and *Leishmania* parasites. By improving knowledge of *Forcipomyia* (*Lasiohelea*) diversity, host-feeding behavior, and parasite associations, this study provides baseline evidence for vector surveillance and supports future investigations into the potential role of *Forcipomyia* (*Lasiohelea*) midges in the circulation of *Leishmania* parasites in Southern Thailand.

## Methods

### Investigation site and *Forcipomyia* midge collection

**Fig. 1:**
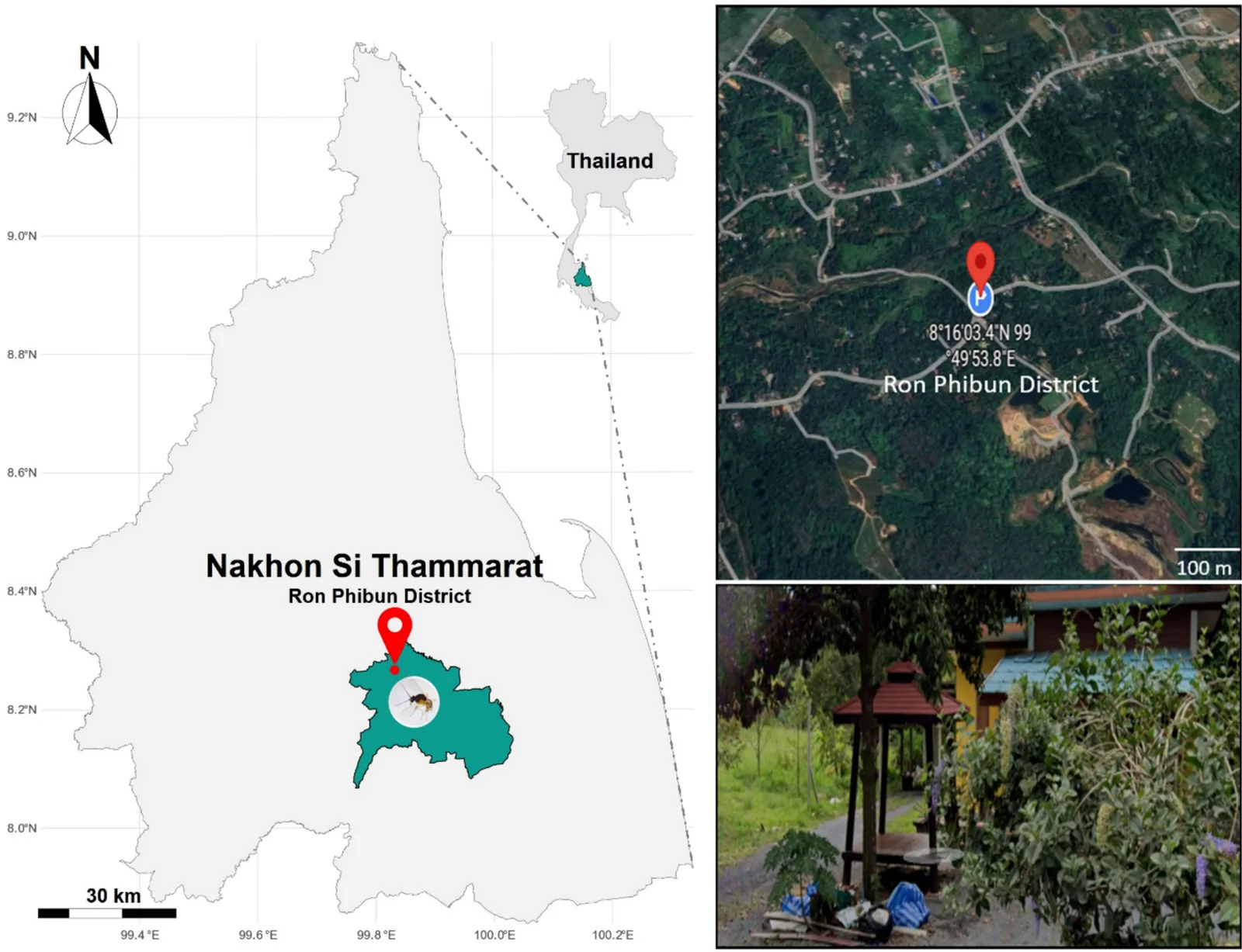
Geographical location of the *Forcipomyia* collection site in Ron Phibun District, Nakhon Si Thammarat Province, Thailand (8°16′03.4″N, 99°49′53.8″E). The outline map of Thailand and Nakhon Si Thammarat Province was generated using RStudio version 4.5.1 [25], while the satellite imagery was adapted from Google Earth (https://earth.google.com/web/search/Thailand)

Adult *Forcipomyia* biting midges were collected near the residence of a patient previously diagnosed with localized cutaneous leishmaniasis in Ron Phibun District, Nakhon Si Thammarat Province, Southern Thailand (Fig. 1) using sweep nets. The collection site was located in a rural residential area surrounded by vegetation and green spaces typical of Southern Thailand. Two domestic dogs and one pet parrot were present at the household, while some nearby households kept free-ranging red junglefowl (*Gallus gallus spadiceus*). Fine-mesh sweep nets, specifically designed for capturing small flying insects, were used to maximize collection efficiency. Sampling was conducted during daylight hours, coinciding with the peak activity of day-feeding midges, between 10:00 a.m. and 4:00 p.m., over three consecutive days for each collection period in January 2025 and June 2025. Weather conditions during sampling were characterized by frequent rainfall in January and intermittent rainfall in June, reflecting the region's typical seasonal patterns. Collectors performed repeated, smooth back-and-forth sweeping motions for 5–10 min per session through the airspace and low-lying vegetation where midges were commonly found. Captured specimens were promptly transferred from the nets into ethanol-filled containers for preservation. All samples were then transported to the Center of Excellence in Vector Biology and Vector-Borne Disease at Chulalongkorn University for downstream analyses.

### Specimen selection and non-destructive DNA extraction

Initially, *Forcipomyia* midges were selected from the specimen collection under a stereomicroscope (EZ4 HD, Leica, Germany) based on morphological characteristics, specifically the presence of the terminal papilla on the antenna [26]. Female specimens were distinguished from males by antennal morphology and the presence of a spermatheca or spermathecae, whereas males were identified by the presence of male genitalia. Subsequently, female specimens exhibiting a single spermatheca were selected for further analysis from the collected *Forcipomyia* samples, as this morphological characteristic is highly distinctive within the genus [26]. The presence of a single spermatheca serves as a preliminary diagnostic feature, facilitating the initial filtering process to identify specimens more likely to belong to the subgenus *Lasiohelea*, which is characterized by a single spermatheca.

To achieve definitive taxonomic identification, an integrated approach, combining detailed morphological assessment and molecular techniques, was employed. First, genomic DNA (gDNA) was extracted from all selected *Forcipomyia* specimens bearing a single spermatheca using a non-destructive protocol as previously described [27], thereby preserving morphological features for further microscopic examination. Briefly, each specimen was digested in 200 µL of lysis buffer containing Proteinase K and incubated at 50 °C overnight. The resulting lysate was purified using a magnetic bead-based system with the GENTi™ Advanced Genomic DNA Extraction Kit (GeneAll^®^, Seoul, South Korea). The DNA concentration and purity were assessed using a NanoDrop 2000c spectrophotometer (Thermo Fisher Scientific, MA, USA). Extracted DNA samples were stored at −20 °C until further species determination and pathogen screening. The post-extraction insect remains were preserved in 70% ethanol at room temperature for subsequent morphological evaluation.

### Mitochondrial *cox*1 amplification and direct Sanger sequencing

Species identification of all selected specimens was performed using conventional PCR amplification of the mitochondrial cytochrome* c* oxidase subunit 1 (*cox*1) gene, with LCO1490 and HCO2198 primers as detailed in Table 1. The PCR reaction mixture contained 4 µL of gDNA, 1.5 µL of each 10 µM primer, 25 µL of 2 × KAPA HiFi HotStart ReadyMix (Roche, Basel, Switzerland), and nuclease-free water to a final volume of 50 µL. Genomic DNA from *Culicoides peregrinus* was used as a positive control to validate the PCR reaction, while nuclease-free water served as the negative control to verify the presence of contamination. Thermocycling conditions consisted of an initial denaturation at 95 °C for 5 min, followed by 40 cycles of denaturation at 98 °C for 30 s, annealing at 46 °C for 30 s, and extension at 72 °C for 1 min, with a final extension at 72 °C for 10 min. PCR products were verified by electrophoresis on a 1.5% (*w*/*v*) agarose gel stained with ethidium bromide (Promega Corporation, WI, USA) together with the GENESTA™ 100 bp DNA ladder marker (GeneAll^®^, Seoul, South Korea) and visualized using the GelDoc Go Imaging System (Bio-Rad, CA, USA). All *cox*1 amplicons were subsequently purified individually using Agencourt AMPure XP beads (Beckman Coulter, CA, USA). The purified samples were subsequently sent to Macrogen, Inc. (Seoul, Korea) for bidirectional Sanger sequencing, using the forward and reverse PCR primers as sequencing primers. The obtained sequences were taxonomically identified by comparing them to reference sequences available in the GenBank database using the Basic Local Alignment Search Tool for nucleotide (BLASTn) (https://blast.ncbi.nlm.nih.gov/Blast.cgi) with parameters optimized for highly similar sequences, as well as through the Barcode of Life Data System (BOLD) (https://www.boldsystems.org/).

**Table 1:** Primer and probe sequences used for molecular detection and identification in this study

Species/gene target	Primer and probe	Oligonucleotide sequence (5′ → 3′)	Amplicon size (bp)	References
*Forcipomyia* spp.			709	[28]
Invertebrate *cox*1 (PCR)	LCO1490	GGTCAACAAATCATAAAGATATTGG		
	HCO2198	TAAACTTCAGGGTGACCAAAAAATCA		
*Leishmania* spp.				
18S rRNA (qPCR)	Le18S-F	CCAAAGTGTGGAGATCGAAG	171	[29]
	Le18S-R	GGCCGGTAAAGGCCGAATAG		
	Le18S probe	6FAM-ACCATTGTAGTCCACACTGC-MGB-NFQ		
ITS1 (PCR)	LITS2	CTGGATCATTTTCCGATGATT	263–280	[30]
	L5.8Sinner	GTTATGTGAGCCGTTATCC		
Vertebrate host species				
Vertebrate *cox*1 (PCR)	VertCOI_7194_F	CGMATRAAYAAYATRAGCTTCTGAY	441	[31]
	Mod_RepCOI_R	TTCDGGRTGNCCRAARAATCA		

### Molecular identification of selected *Forcipomyia* specimens through phylogenetic analysis of *cox*1 sequences and species delimitation tests

To achieve precise species assignment and clarify species boundaries among *Forcipomyia* subgenera, an integrative approach combining phylogenetic and species delimitation analyses was employed. The *cox*1 sequences obtained from *Forcipomyia* specimens in this study were aligned with 56 representative *Forcipomyia* reference sequences available in GenBank using ClustalW implemented in BioEdit version 7.2.6 [32]. The aligned sequences were subjected to phylogenetic analyses using both Bayesian inference (BI) and maximum likelihood (ML) methods. For these analyses, *cox*1 sequences of *Fletcherimyia abdita* (GenBank: OR381187 and OR381196) were selected as outgroup taxa to root the phylogenetic trees.

For BI, the best-fitting evolutionary model was determined using ModelFinder [33], which identified the GTR + F + I + G4 model as the one with the lowest Bayesian Information Criterion (BIC). A Bayesian phylogenetic tree was then generated using BEAST version 2.7.7 [34], with 100 million Markov Chain Monte Carlo (MCMC) steps performed, discarding the first 25% as “burn-in” to estimate posterior probabilities robustly.

A maximum likelihood tree was constructed using MEGA11 [35], employing the General Time Reversible model with discrete gamma distribution and invariant sites (GTR + G + I), determined as the optimal substitution model. Bootstrap values were calculated from 1000 replicates to assess branch support. Final trees were visualized using FigTree version 1.4.4 [36] for evaluating phylogenetic relationships and species delimitations within the *Forcipomyia* genus.

Species delimitation analyses were performed on the *Forcipomyia cox*1 sequence dataset to define species boundaries using two complementary methods: Assemble Species by Automatic Partitioning (ASAP) [37] through iTaxoTools (https://itaxotools.org/) and multi-rate Poisson Tree Processes (mPTP) analysis [38] (https://mptp.h-its.org/#/tree). For the ASAP method, three substitution models were tested: simple distance (p-distances), Jukes-Cantor (JC69), and Kimura 2-parameter (K80). The partition with the lowest score was considered the most reliable species delimitation. Nucleotide sequences from specimens identified as *Forcipomyia* (*Lasiohelea*) were submitted to GenBank.

### Morphological assessment of molecularly identified *Forcipomyia* (*Lasiohelea*) specimens

Following DNA extraction and molecular identification, the remaining insect structures were carefully mounted on microscope slides in Hoyer’s medium and incubated at 37 °C for two weeks to enable detailed morphological examination. Key morphological features, including mandibles, maxillary palp, cibarium, spermatheca, and wings, were systematically observed, visualized, and photographed with an Olympus CX43 light microscope equipped with an Olympus SC180 digital camera (Olympus, Tokyo, Japan). Morphological identification was conducted using taxonomic keys and published descriptions specific to Asian *Forcipomyia* (*Lasiohelea*) midges [26, 39–42]. Specimens classified within the *Lasiohelea* clade based on molecular phylogenetics underwent thorough morphological verification to assess congruence. Additionally, specimens excluded from the *Lasiohelea* clade by molecular analysis were also examined morphologically to confirm or refine their taxonomic placement.

### Initial screening of *Leishmania* spp. DNA using 18S rRNA-qPCR

Genomic DNA samples identified as *Forcipomyia* (*Lasiohelea*) through integrative molecular and morphological analyses were initially screened for *Leishmania* DNA using a TaqMan quantitative PCR (qPCR) assay targeting a conserved region of the 18S ribosomal RNA (18S rRNA) gene, as shown in Table 1. The PCR reaction was prepared in a total volume of 20 µl, comprising 10 µl of TaqMan™ Fast Advanced Master mix (Thermo Scientific, MA, USA), 0.5 µM of each primer from a 10 µM stock, 0.5 µl of a 10 µM probe, and 2 µl of gDNA. Primer and probe sequences are detailed in Table 1. Amplifications were performed on a QuantStudio™ 5 Real-Time PCR System (Applied Biosystems, CA, USA) under the following cycling conditions: initial denaturation at 95 °C for 2 min, followed by 45 cycles of denaturation at 95 °C for 15 s and annealing/extension at 60 °C for 15 s. A cycle threshold (Ct) value below 40 was interpreted as positive for *Leishmania* DNA.

### Amplification of *Leishmania* DNA by conventional ITS1-PCR

The gDNA samples of *Forcipomyia* (*Lasiohelea*) were also used as templates for conventional PCR targeting the internal transcribed spacer-1 (ITS1) region to confirm the qPCR results and provide complementary species identification, due to the variability of ITS1 sequences across *Leishmania* species. Amplification was carried out using primers LITSR2 and L5.8Sinner, as detailed in Table 1, which produce an amplicon of approximately 263–280 bp. The PCR reaction mixture consisted of 4 µL of genomic DNA, 1.5 µL of each 10 µM primer, 25 µL of 2 × KAPA HiFi HotStart ReadyMix (Roche, Basel, Switzerland), and nuclease-free water to a final volume of 50 µL. Thermal cycling conditions included an initial denaturation at 95 °C for 5 min, followed by 35 cycles of denaturation at 98 °C for 30 s, annealing at 53 °C for 30 s, and extension at 72 °C for 30 s with a final extension step at 72 °C for 10 min. Genomic DNA extracted from *L. martiniquensis* promastigotes (MHOM/TH/2022/CULE6) was used as a positive control, whereas nuclease-free water was used as a negative control. PCR products were verified on a 1.5% (*w*/*v*) agarose gel stained with ethidium bromide and visualized as previously described. Positive ITS1 amplicons were purified for downstream amplicon sequencing to identify *Leishmania* species.

### Taxonomic profiling of *Leishmania* using nanopore-based metabarcoding

To investigate whether multiple *Leishmania* species are present in each genomic DNA sample, nanopore-based metabarcoding using MinION^®^ ITS1 sequencing was employed. Purified ITS1 amplicons were quantified with the Qubit™ dsDNA High Sensitivity Assay Kit (Thermo Fisher Scientific, MA, USA). Subsequently, the amplicons underwent end repair using the NEBNext^®^ Ultra™ II End Repair/dA-Tailing Module (New England Biolabs, MA, USA) and were ligated to native barcodes from the Native Barcoding Kit 96 V14 (Cat. No. SQK-NBD114.96, Oxford Nanopore Technologies, UK), using the NEB Blunt/TA Ligase Master Mix (New England Biolabs, MA, USA). All barcoded amplicons were pooled and prepared for adaptor ligation with the NEBNext Quick Ligation Module (New England Biolabs, MA, USA). Following purification with AMPure magnetic beads, 50 fmol of the final library was loaded onto a MinION^®^ R10.4.1 flow cell for overnight sequencing. Base calling was performed using the super-accuracy model (dna_r10.4.1_e8.2_400bps_sup@v.4.3.0 model) in Dorado version 7.3.11. Reads with quality scores below 20 were removed by Chopper version 0.7.0 [43]. Using amplicon_sorter.py (version 2024_02_20) [44], consensus sequences were generated from high-quality reads with a size selection of 200–350 bp. To ensure reliable consensus sequences, clusters required a minimum coverage of 100 reads and represented at least 5% of the total reads per sample. This abundance threshold filters out low-frequency clusters likely resulting from sequencing errors, contamination, or artifacts, while preserving detection sensitivity for biologically relevant minor variants. Clusters that failed to meet these criteria were excluded from further analysis, balancing error reduction with the detection of minor species. The obtained ITS1 consensus sequences were aligned against reference sequences in GenBank using Megablast, with optimized settings for highly similar sequences. Species-level taxonomic assignment of *Leishmania* was established at a sequence similarity of 98% or higher. The resulting *Leishmania* ITS1 sequences were then deposited in GenBank.

### Phylogenetic analysis of *Leishmania* ITS1 sequences

To confirm the BLASTn identification of *Leishmania* species, *ITS1* sequences amplified from infected humans, including the patient in this study, and *Forcipomyia* (*Lasiohelea*) specimens were aligned alongside 37 reference sequences from GenBank representing various *Leishmania* species and geographic origins. The ITS1 sequence of *Crithidia mellificae* (GenBank: KJ722747) was used as the outgroup for both BI and ML phylogenetic analyses. For BI, the HKY + F + I model was selected as the best-fitting model based on the lowest Bayesian Information Criterion (BIC), and a Bayesian tree was constructed using BEAST version 2.7.7 [34], running 100 million MCMC steps with the first 25% discarded as “burn-in”. Simultaneously, MEGA11 [35] was used to construct a maximum likelihood tree with 1000 bootstrap replicates, employing the K2 + I model identified as optimal. The resulting BI and ML trees were visualized with FigTree version 1.4.4 [36] to assess phylogenetic relationships.

### Blood meal source identification through vertebrate *cox*1-PCR and nanopore-based metabarcoding

The extracted genomic DNA from an engorged *Forcipomyia* specimen was analyzed to identify its blood meal source using PCR targeting vertebrate mitochondrial *cox*1 with primers VertCOI_7194_F and Mod_RepCOI_R, as detailed in Table 1. PCR reactions were conducted in a 50 µL total volume containing 25 µL of 2 × KAPA HiFi HotStart ReadyMix, 1.5 µL of each 10 µM primer, and 4 µL of gDNA template. Thermal cycling conditions included an initial denaturation at 95 °C for 3 min, followed by 40 cycles of 95 °C for 40 s, annealing at 48.5 °C for 30 s, extension at 72 °C for 1 min, and a final extension at 72 °C for 7 min. Cow genomic DNA served as a positive control, and nuclease-free water was used as a negative control. The amplicon sample was purified using Agencourt AMPure XP beads (Beckman Coulter, CA, USA) and subsequently subjected to native barcode sequencing on the MinION^®^ platform following established protocols. Basecalling employed a super-accuracy model, followed by consensus sequence analysis from high-quality reads with a size selection of 300–500 bp using amplicon_sorter.py [44]. For taxonomic identification, the consensus sequence obtained was compared to GenBank reference sequences using BLASTn.

## Results

### Sample collection and preliminary selection based on the presence of a single spermatheca

A total of 264 *Forcipomyia* midges were collected from the study area, comprising 255 females and nine males. Among the females, 72 possessed a single spermatheca, whereas 183 had two spermathecae. Only females with a single spermatheca (*n* = 72) were selected for further morphological and molecular analyses. The blood-fed specimen (NST_14), which also possessed a single spermatheca, was included in this subset and was readily identified as a *Forcipomyia* (*Lasiohelea*) midge based on the presence of a blood meal in the abdomen.

### DNA barcoding and phylogenetic analyses

Seventy-two female specimens, each possessing a single spermatheca, were analyzed using *cox*1-PCR and direct Sanger sequencing. Species identification of these specimens was conducted by comparing the obtained sequences against reference databases, including BOLD and GenBank. Of the 72 *cox*1 sequences, 27 were identified at the family level as Ceratopogonidae sp., with similarity scores ranging from 99.69% to 100%. The remaining 45 sequences were identified at the genus level as *Forcipomyia* sp. in the BOLD database, with similarity scores ranging from 91.31% to 100%. Meanwhile, BLASTn analysis assigned 62 sequences to the family Ceratopogonidae, with percent identities spanning 86.05% to 98.30%. The remaining ten sequences matched available reference sequences at the species level, including *Forcipomyia* (*Euprojoannisia*) *fuscimana* (GenBank: MZ539943) (*n* = 3; 98.29% for NST103 and NST_111 and 98.46% for NST_82), *Forcipomyia* (*Lasiohelea*) *parvitas* (GenBank: OM632702) (*n* = 2; 98.32% for NST_28 and 98.88% for NST_102), *Forcipomyia* (*Lasiohelea*) *peditata* (GenBank: OR117382) (*n* = 2; 91.31% for NST_1 and NST_14), *Forcipomyia* (*Lasiohelea*) *humilavolita* (GenBank: PP199400) (*n* = 2; 87.98% for NST_38 and 88.12% for NST_44), and *Forcipomyia* (*Lasiohelea*) *taiwana* (GenBank: KF528690) (*n* = 1; 88.82% for NST_12). As shown in Table S1, the identity score values varied across these sequences, with some indicating strong matches reflecting closer genetic relationships, while others showed relatively low similarity, suggesting poor matches.

**Fig. 2:**
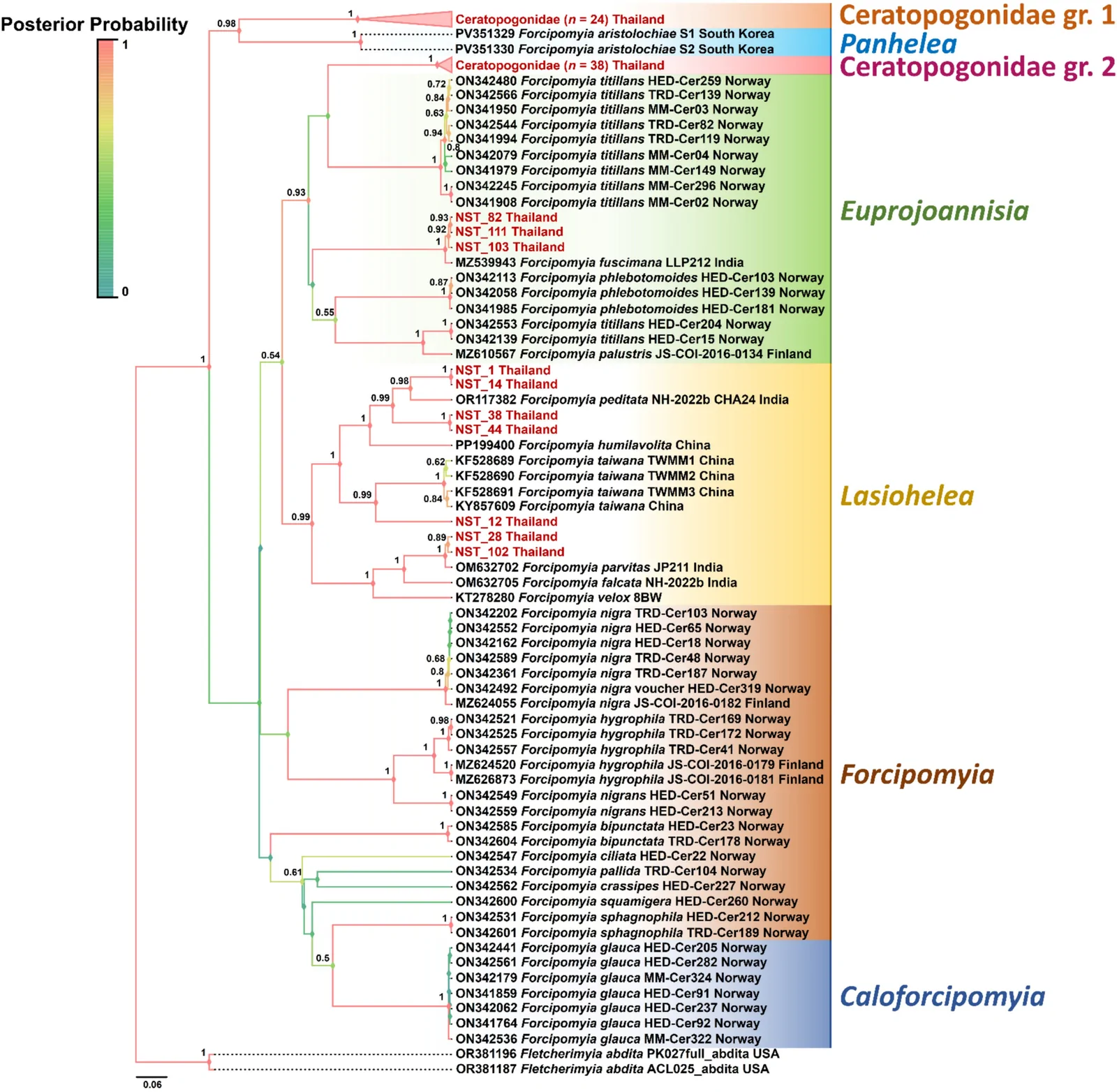
Bayesian phylogenetic tree of *Forcipomyia* species inferred from *cox*1 gene sequences. Posterior probabilities are indicated at the nodes, represented by a color spectrum ranging from 0 (blue green) to 1 (red). Sequences newly generated in this study are highlighted in bold red text. Major subgenera and genera within Ceratopogonidae are color coded: Ceratopogonidae groups 1 and 2 (light brown and violet) represent specimens that could not be confidently assigned to any subgenus, *Panhelea* (light blue), *Euprojoannisia* (green), *Lasiohelea* (orange), *Forcipomyia* (brown), and *Caloforcipomyia* (blue). The scale bar represents 0.06 substitutions per site

**Fig. 3:**
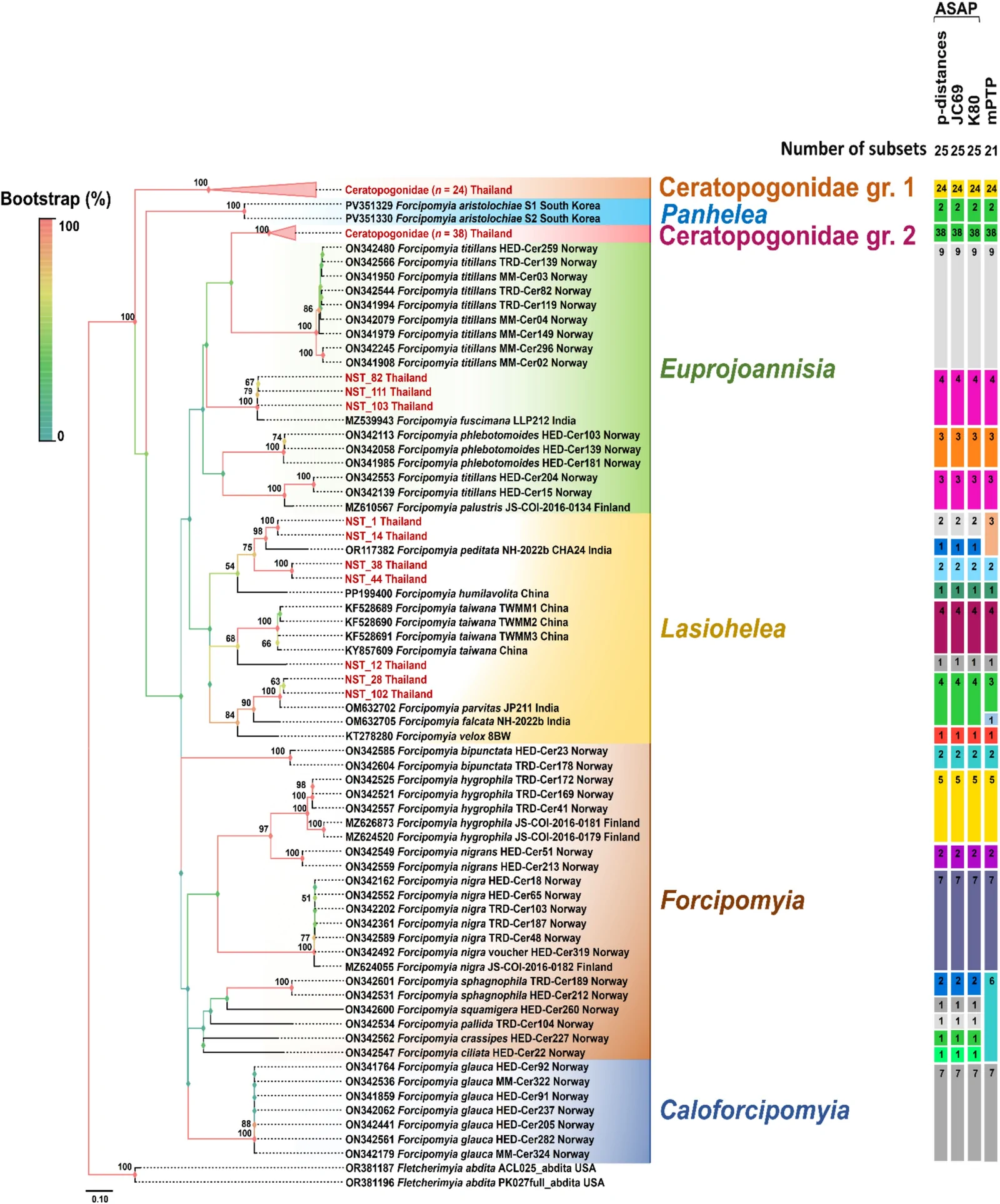
Maximum likelihood phylogenetic tree of *Forcipomyia* species inferred from *cox*1 gene sequences. Bootstrap support values (%) are shown at the nodes, represented by a color spectrum ranging from 0 (blue green) to 100 (red). Newly generated Thai sequences are highlighted in bold red. Major clades are shaded according to their taxonomic groups, consistent with the Bayesian tree (Fig. 2). Species delimitation analyses (ASAP and mPTP) revealed congruent species boundaries with the ML topology, supporting distinct lineages within the *Lasiohelea* clade, particularly among Thai specimens. The scale bar represents 0.10 substitutions per site

The phylogenetic relationships of 72 sequences identified in this study were reconstructed using both BI and ML methods, yielding largely congruent topologies that support the robustness of the inferred clades. The BI tree, evaluated through posterior probabilities, exhibited high statistical support at most nodes, with many key branches supported by values ≥ 0.90 and several nodes receiving full support (1.0) (Fig. 2). The ML analysis provided bootstrap support mainly at subclades and lineages, with several internal branches exhibiting values ≥ 90% (Fig. 3).

Both phylogenetic trees consistently recovered major known taxonomic groups, including *Panhelea*, *Euprojoannisia*, *Lasiohelea*, *Forcipomyia*, and *Caloforcipomyia*. Additionally, two highly supported clusters, designated as Ceratopogonidae groups 1 and 2, comprised specimens that could not be confidently assigned to any existing subgenus, highlighting potential novel or unresolved lineages. The basal position of Ceratopogonidae group 1 and *Panhelea* was strongly supported, indicating early divergence events within the family. Within *Euprojoannisia*, Thai specimens NST_82, NST_103, and NST_111 formed a well-supported and distinct subclade (Bayesian inference posterior probability (PP) = 0.92–1; maximum likelihood bootstrap support (BS) = 67–100%), suggesting a potentially novel lineage in the region.

Among *Lasiohelea*, seven Thai specimens (NST_1, NST_12, NST_14, NST_28, NST_38, NST_44, and NST_102) were grouped in the same clade but divided into multiple well-defined clusters rather than forming a single cohesive lineage. The first cluster, comprising NST_1 and NST_14, grouped closely with *F.* (*L.*) *peditata* from India (GenBank: OR117382) with strong statistical support (PP = 0.98–1; BS = 98–100%), indicating a close evolutionary relationship. The second cluster, including NST_38 and NST_44, was placed near *F.* (*L.*) *humilavolita* from China (GenBank: PP199400). The third cluster, including NST_12, grouped with *F.* (*L.*) *taiwana* from China (GenBank: KF528689-KF528691 and KY857609). The fourth cluster, comprising NST_28 and NST_102, grouped closely with *F.* (*L.*) *parvitas* from India (GenBank: OM632702) with robust support (PP = 0.89–1; BS = 63–100%) and shared the clade with *Forcipomyia* (*Lasiohelea*) *falcata* (GenBank: OM632705) and *Forcipomyia* (*Lasiohelea*) *velox* (GenBank: KT278280). Relatively long branch lengths separating these clusters, combined with strong internal support, indicate substantial genetic divergence between groups, alongside limited variation within each cluster, reflecting distinct but closely related taxonomic entities within the *Lasiohelea* complex. Sequences of *Forcipomyia* (*Lasiohelea*) specimens from this study were deposited in GenBank under accession numbers PX502056–PX502062.

Species delimitation using both ASAP and mPTP methods aligned well with this phylogenetic framework, identifying 25 and 21 putative species clusters, respectively. Notably, the cluster of NST_82, NST_103, and NST_111 exhibited a short branch length separating it from *F.* (*E.*) *fuscimana*, supported by both delimitation tests and corroborated by BLASTn results showing over 98% sequence identity, confirming conspecificity within the *Euprojoannisia* clade. Conversely, the three clusters NST_1 and NST_14; NST_38 and NST_44; and NST_12 showed long branch lengths and were supported by delimitation methods as distinct from *F.* (*L.*) *peditata*, *F.* (*L.*) *humilavolita*, and *F.* (*L.*) *taiwana*, respectively. Lastly, the cluster of NST_28 and NST_102 exhibited a short branch length, close to *F.* (*L.*) *parvitas*, and was supported by delimitation and BLASTn analyses, with identities exceeding 98%, confirming the same species status. These findings highlight the utility of integrating molecular species delimitation, phylogenetic inference, and sequence similarity data to delineate species boundaries and reveal cryptic genetic diversity among *Forcipomyia* midges in Thailand.

### Morphological characterization of the *Lasiohelea* and non-*Lasiohelea* groups

**Fig. 4:**
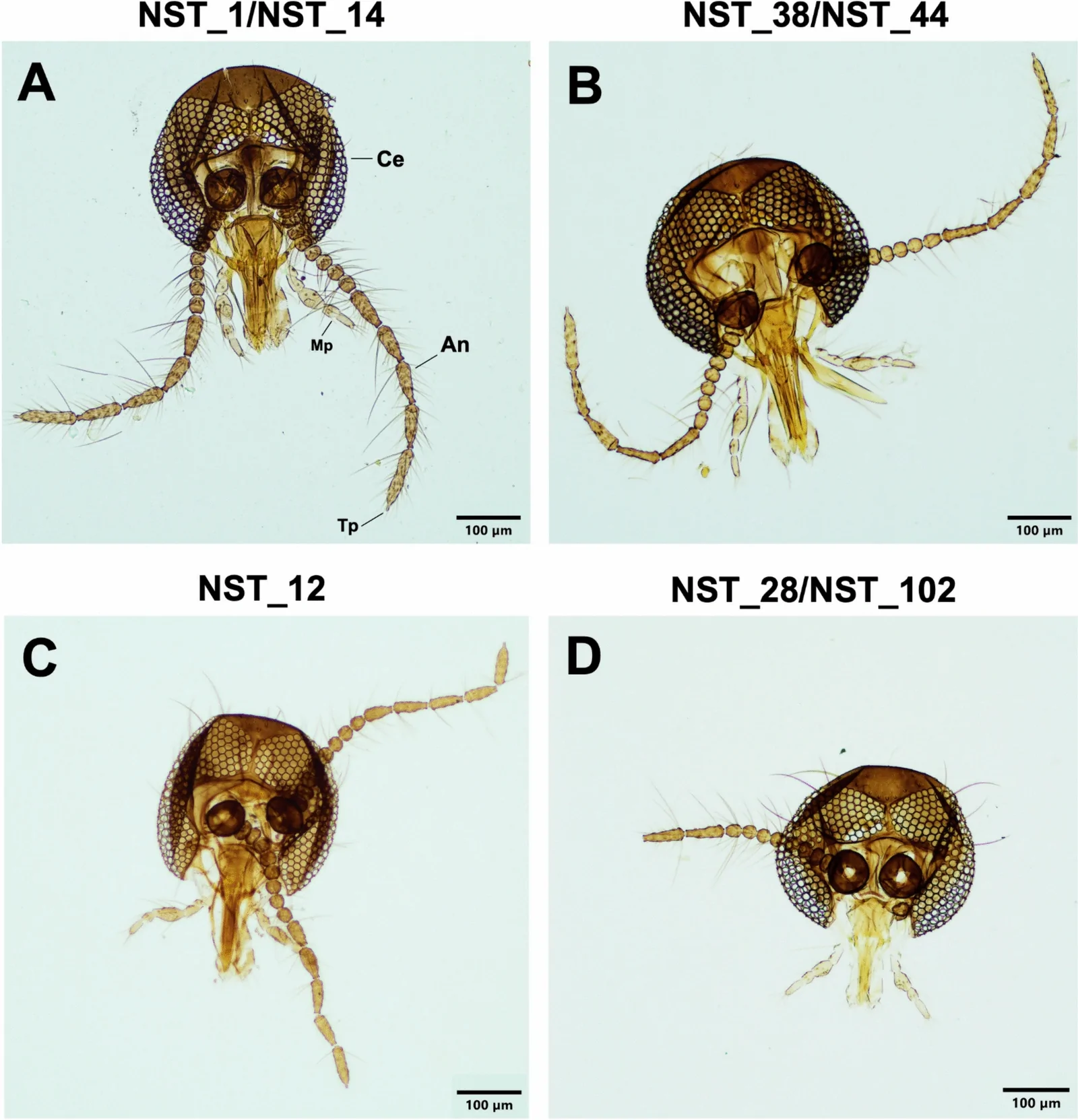
Head morphology of *Lasiohelea* specimens collected in this study. Frontal views of the head capsule and mouthparts under compound microscopy are shown for specimens NST_1 and NST_14 (**A**), NST_38 and NST_44 (**B**), NST_12 (**C**), and NST_28 and NST_102 (**D**). An, Antenna; Ce, Compound eye; Mp, Maxillary palpus; Tp, Terminal papilla

**Fig. 5:**
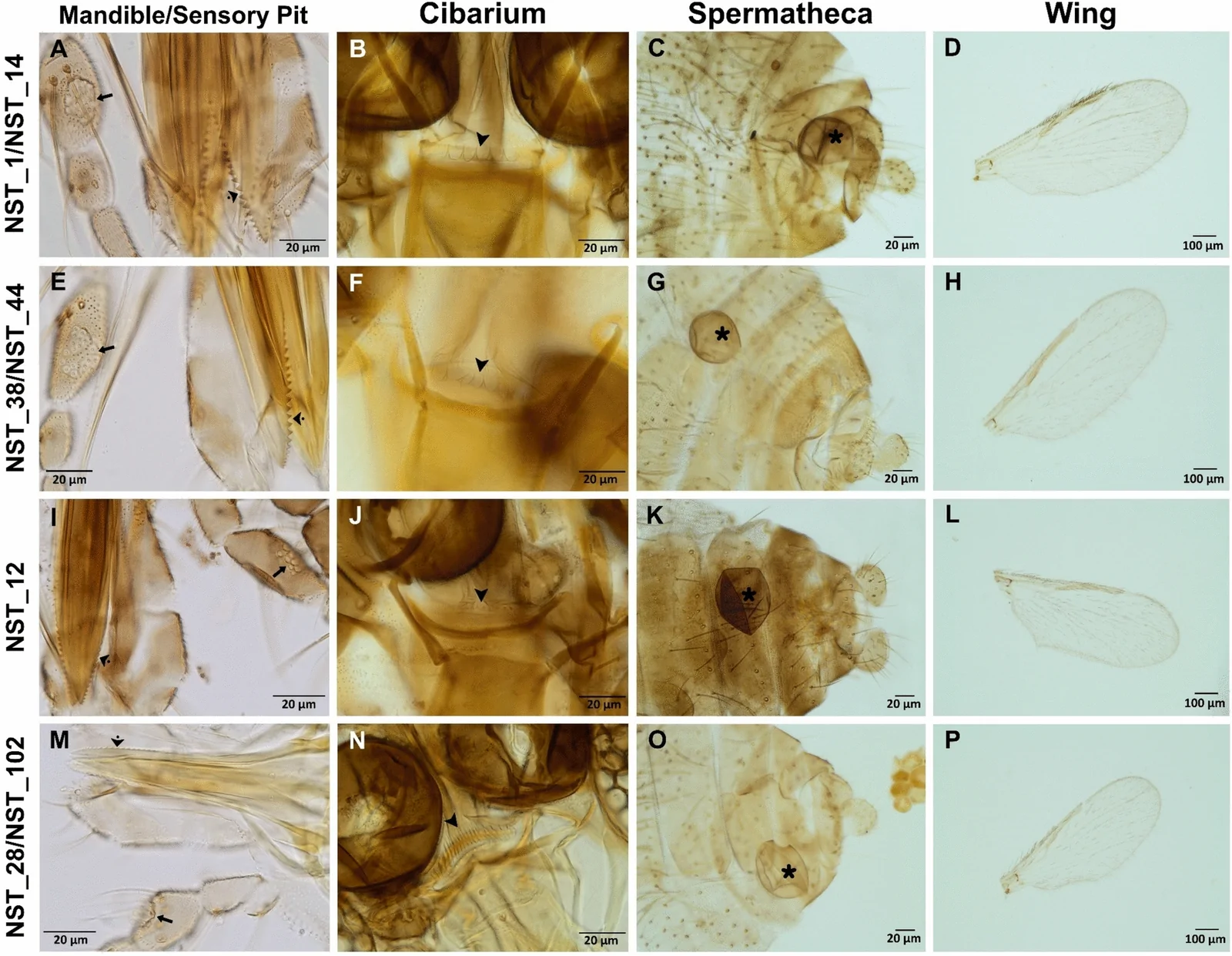
Comparative morphological features of *Lasiohelea* specimens identified in this study. **A**–**D** show NST_1/NST_14, **E**–**H** NST_38/NST_44, **I**–**L** NST_12, and **M**–**P** NST_28/NST_102. Each panel displays the mandible and sensory pit (**A**, **E**, **I**, **M**), cibarium (**B**, **F**, **J**, **N**), spermatheca (**C**, **G**, **K**, **O**), and wing (**D**, **H**, **L**, **P**). Circle arrows denote mandibular teeth; arrows highlight the sensory pit; arrowheads indicate the cibarial armature; asterisks mark the spermatheca

The *Lasiohelea* specimens analyzed in this study exhibited distinct morphological variation that closely corresponds to four molecularly defined groups, each exhibiting characteristic features of the subgenus *Lasiohelea*. Frontal microscopic views of the head capsule and mouthparts for these specimens are shown in Fig. 4. Specimens NST_1 and NST_14, phylogenetically closest to *F.* (*L.*) *peditata*, exhibited some divergence in sequence percent identity compared to *F.* (*L.*) *peditata*; however, their morphological features aligned well with those described for this species [34], including mandibles with 21 visible teeth, a greatly inflated third maxillary palp segment bearing a well-developed, large sensory pit at the midpoint with 16 capitate sensilla (Fig. 5A), a cibarium with six teeth (Fig. 5B), a subglobular spermatheca without a neck (Fig. 5C), and wings with costa extending beyond the middle of the wing and an elongated second radial cell densely covered with macrotrichia, especially along the costa and wing margin (Fig. 5D).

Specimens NST_38 and NST_44, genetically related to *F.* (*L.*) *humilavolita*, also showed some divergence in sequence percent identity compared to *F.* (*L.*) *humilavolita*; however, their morphological features closely matched those of this species [26], including mandibles with 19–22 visible teeth, an inflated third maxillary palp segment with a well-developed, large sensory pit containing 20–21 capitate sensilla (Fig. 5E), a cibarium with 9–10 sharp teeth (Fig. 5F), a slightly rounded spermatheca lacking a neck (Fig. 5G), and wings with costa extending beyond the middle and an elongated second radial cell bearing evenly distributed macrotrichia (Fig. 5H).

Specimen NST_12, affiliated phylogenetically with *F.* (*L.*) *taiwana*, featured mandibles with 20 min teeth, an inflated third maxillary palp segment with a well-developed sensory pit at the midpoint containing 18 capitate sensilla (Fig. 5I), a cibarium with 11 sharp teeth (Fig. 5J), an ovoid to subglobular spermatheca without a neck (Fig. 5K), and wings with costa extending beyond the middle and an elongated second radial cell with macrotrichia along the costa (Fig. 5L).

Lastly, specimens NST_28 and NST_102, genetically close to *F.* (*L.*) *parvitas*, possessed mandibles with 19–20 tiny teeth and an inflated third maxillary palp segment with a well-developed sensory pit containing capitate sensilla. However, the exact number of sensilla could not be determined due to incomplete visibility (Fig. 5M). They displayed a cibarium with 22–23 fine, sharp teeth (Fig. 5N), an ovoid to subglobular spermatheca without a neck (Fig. 5O), and wings with costa extending beyond the middle and an elongated second radial cell with macrotrichia mainly along the costa (Fig. 5P). Despite the high sequence similarity to *F.* (*L.*) *parvitas*, the lack of a detailed morphological description of the female of this species currently precludes confirming morphological concordance with known *F.* (*L.*) *parvitas* females.

Overall, the *Lasiohelea* specimens exhibited clear and well-defined morphological divergence across these four groups, particularly in mandibular dentition, cibarial armature, spermatheca shape, and subtle variations in wing macrotrichia distribution. These distinctive morphological distinctions closely corresponded with phylogenetic groupings and species delimitations inferred from *cox*1 data, supporting their status as distinct but related lineages within the *Lasiohelea* complex.

**Fig. 6:**
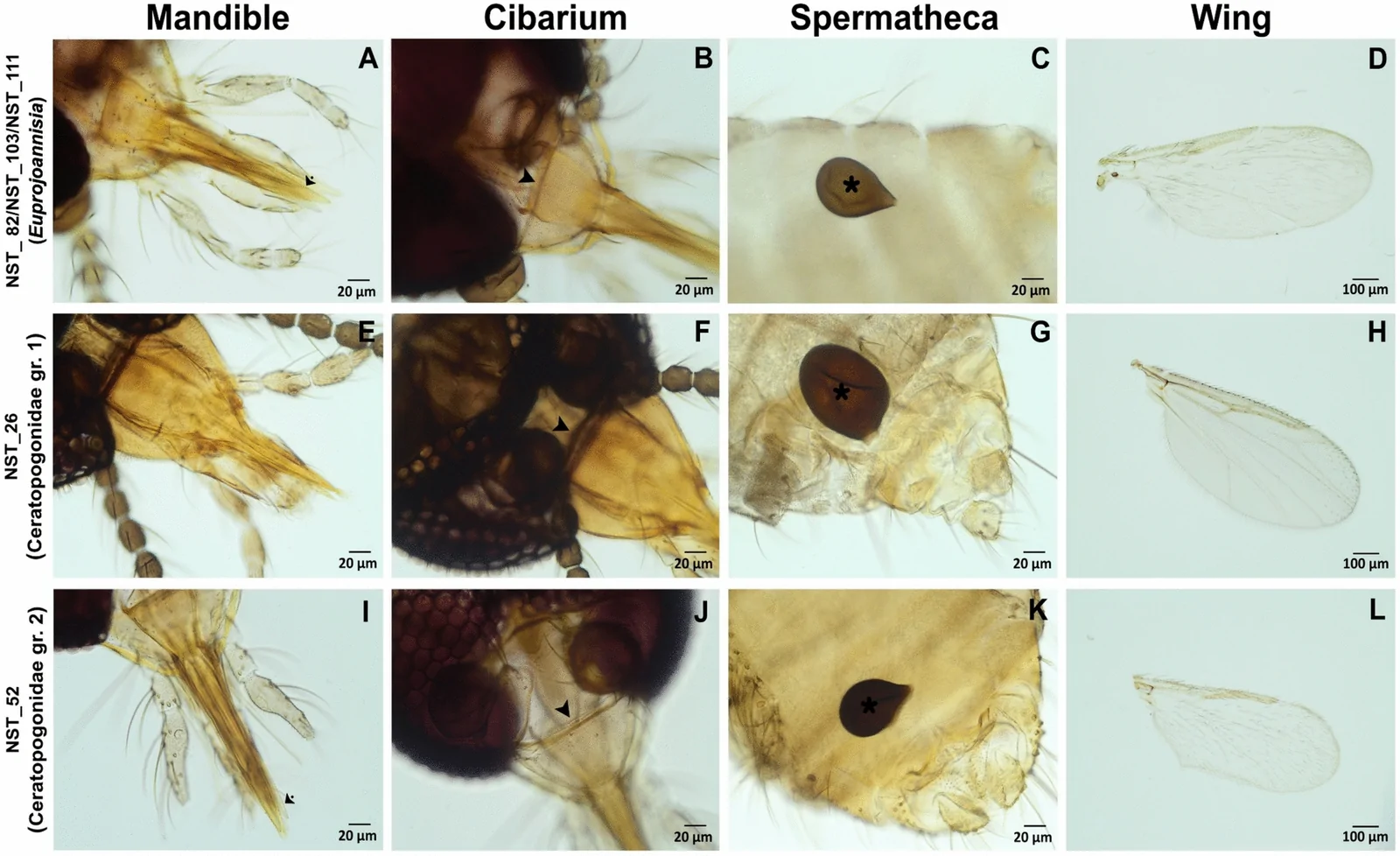
Representative morphological features of non-*Lasiohelea* specimens identified in this study. **A**–**D** show NST_82/NST_103/NST_111 (*Euprojoannisia*), **E**–**H** NST_26 (Ceratopogonidae group 1), and **I**–**L** NST_52 (Ceratopogonidae group 2). Circle arrows indicate minute mandibular teeth; arrowheads indicate the unarmed cibarium lacking visible teeth; asterisks mark the spermatheca

In contrast, non-*Lasiohelea* specimens, assigned to the species of the subgenus *Euprojoannisia* and two additional Ceratopogonidae lineages, exhibit markedly distinct morphology consistent with their molecular placements. Specimens NST_82, NST_103, and NST_111, closely related to *F.* (*E.*) *fuscimana*, possessed mandibles with minute teeth, a less-developed sensory pit (Fig. 6A), and an unarmed cibarium (Fig. 6B). Their spermatheca is single, ovoid with a distinct neck, and pigmented (Fig. 6C). Their wings displayed sparse microtrichia with the costa extending approximately to the middle (Fig. 6D). These characters are consistent with the female morphology described for members of the subgenus *Euprojoannisia* [45]. Additionally, Ceratopogonidae groups 1 and 2, represented by specimens NST_26 and NST_52, exhibited barely visible or indistinct mandibular dentition, an unarmed cibarium, a pigmented ovoid spermatheca with a neck, and wings with sparse macrotrichia and a costa extending beyond the middle of the wing (Fig. 6E–L).

Consequently, these morphological traits clearly differentiate the *Lasiohelea* complex from non-*Lasiohelea* taxa. The *Lasiohelea* group features well-defined mandibular teeth and a robust cibarial armature. In contrast, non-*Lasiohelea* specimens, including those from *Euprojoannisia﻿* and other Ceratopogonidae lineages, typically show reduced mandibular dentition and an unarmed cibarium. This simpler morphology in non-*Lasiohelea* groups reflects their evolutionary divergence from the *Lasiohelea* complex. These morphological differences, therefore, highlight important taxonomic distinctions and support molecular evidence for genetically distinct lineages within the genus *Forcipomyia*.

Taken together, the integrative morphological and molecular evidence from the present study resolved four *Forcipomyia* (*Lasiohelea*) taxa from Southern Thailand, comprising *F.* (*L.*) *parvitas* and three putatively distinct lineages closely related to *F.* (*L.*) *peditata*, *F.* (*L.*) *humilavolita*, and *F.* (*L.*) *taiwana*. In addition, *F.* (*E.*) *fuscimana* and two unresolved Ceratopogonidae lineages were also recovered from specimens initially selected based on the presence of a single spermatheca. These findings provide the first molecularly supported overview of *Forcipomyia* diversity in this leishmaniasis-endemic area.

### Molecular detection and identification of *Leishmania* species in *Lasiohelea* specimens

Of seven *Forcipomyia* (*Lasiohelea*) specimens tested, *Leishmania* 18S rRNA-qPCR yielded positive results in six individuals (NST_1, NST_12, NST_28, NST_38, NST_44, and NST_102), while the blood-fed specimen NST_14 was negative. All qPCR-positive samples were subsequently amplified using conventional ITS1-PCR, and species identification was further achieved through nanopore-based ITS1 metabarcoding. After quality filtering, 55,835 high-quality ITS1 reads were recovered from ITS1-positive samples and processed using amplicon_sorter.py, resulting in 12 high-confidence consensus sequences. These sequences were assigned to *Leishmania* taxa by BLASTn comparison against GenBank reference sequences, as summarized in Table 2.

**Table 2:** Detection of *Leishmania* spp. in *Forcipomyia* (*Lasiohelea*) midges from Thailand based on *Leishmania* 18S rRNA-qPCR and ITS1-PCR followed by MinION^®^ sequencing

Sample code	Species	*Leishmania* spp. 18S rRNA-qPCR	*Leishmania* spp. ITS1-PCR and MinION^®^ sequencing					
			BLASTn result	Closet GenBank reference sequences	No. assigned reads	Sequence length (bp)	Relative abundance (%)	Percent Identity
NST_1	*F.* (*Lasiohelea*) sp.	Positive	*L. martiniquensis*	GQ293226	9112	280	76.3	100
			*L. orientalis*	KY982664	1979	270	16.6	98.9
			*L. major*	MZ502962	847	270	7.1	100
NST_12	*F.* (*Lasiohelea*) sp.	Positive	*L. orientalis*	KY982664	5124	273	65.8	99.6
			*L. amazonensis*	DQ182536	2662	263	34.2	100
NST_28	*F.* (*Lasiohelea*) *parvitas*	Positive	*L. orientalis*	KY982664	4760	272	45.3	99.6
			*L. martiniquensis*	MK603826	4123	278	39.3	98.6
			*L. amazonensis*	DQ182536	1617	263	15.4	99.6
NST_38	*F.* (*Lasiohelea*) sp.	Positive	*L. martiniquensis*	GQ293226	2645	280	100	99.6
NST_44	*F.* (*Lasiohelea*) sp.	Positive	*L. martiniquensis*	GQ293226	13430	280	94.6	100
			*L. orientalis*	OR135533	769	268	5.4	98.9
NST_102	*F.* (*Lasiohelea*) *parvitas*	Positive	*L. martiniquensis*	GQ293226	8767	280	100	100

Both autochthonous species (*L.* (*Mundinia*) *martiniquensis* and *L.* (*Mundinia*) *orientalis*) and non-autochthonous species (*L.* (*Leishmania*) *amazonensis* and *L.* (*Leishmania*) *major*) were detected in *Forcipomyia* (*Lasiohelea*) specimens. Mixed-species detections were identified in four specimens (NST_1, NST_12, NST_28, and NST_44), with some specimens harboring up to three *Leishmania* species concurrently. Notably, *L. martiniquensis* and *L. orientalis* were the most frequently detected taxa across samples, whereas *L. amazonensis* and *L. major* were detected at comparatively lower relative abundances, as detailed in Table 2. The *Leishmania* ITS1 sequences generated in this study were deposited in GenBank under accession numbers PX626647–PX626658.

### Species reaffirmation of *Leishmania* parasites by ITS1 phylogeny

**Fig. 7:**
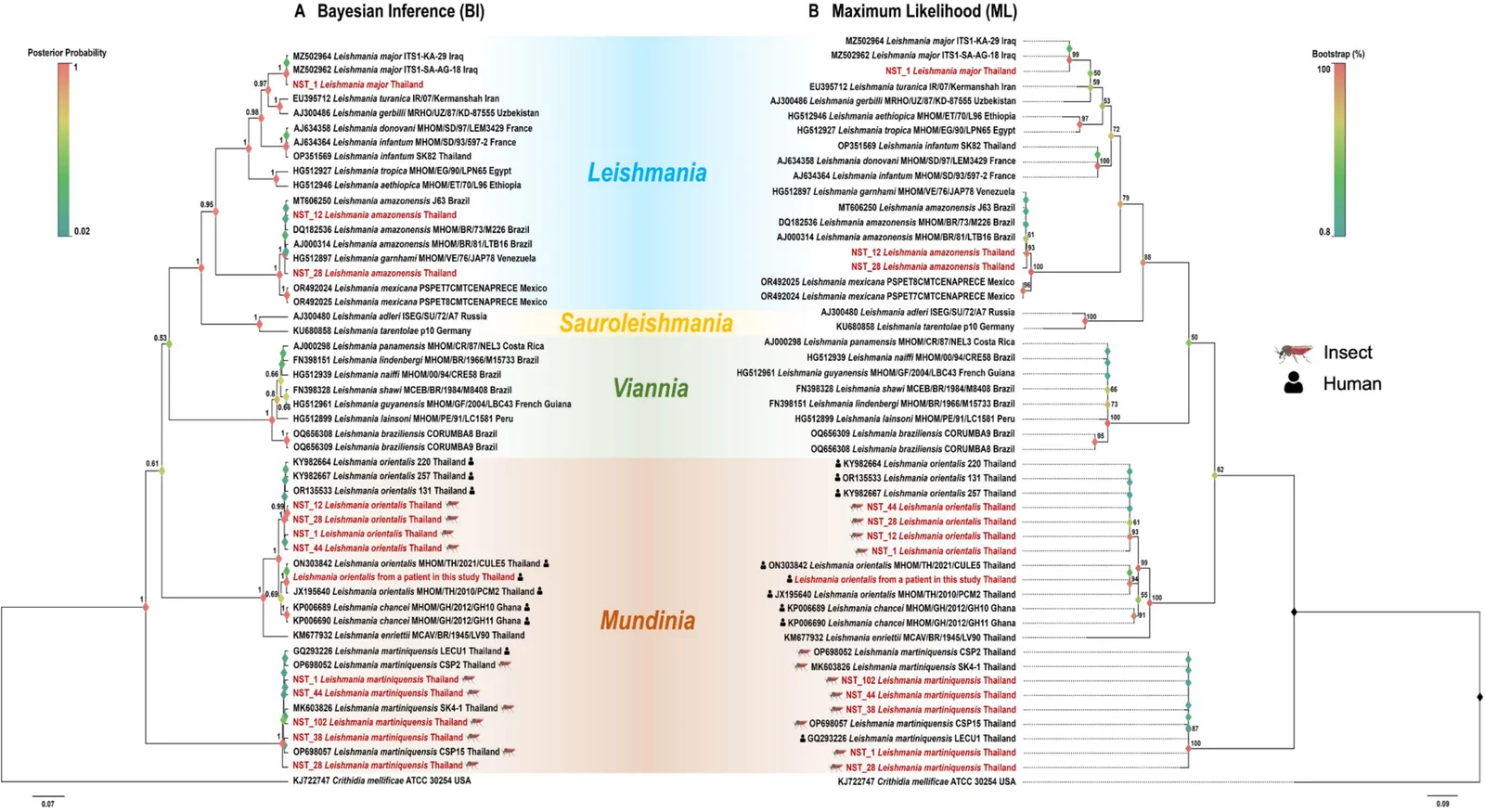
Phylogenetic trees of *Leishmania* spp. based on ITS1 sequences, constructed using Bayesian inference (BI) (**A**) and maximum likelihood (ML) (**B**). Both analyses consistently resolved four major clades (*Leishmania*, *Sauroleishmania*, *Viannia*, and *Mundinia*) with strong statistical support indicated by posterior probabilities and bootstrap values at each node. Sequences from this study, including those derived from *Forcipomyia* (*Lasiohelea*) specimens in Thailand, are highlighted in red with an insect symbol (), while human-derived sequences are marked by a person symbol (). Newly generated sequences of *L. martiniquensis* and *L. orientalis* cluster within the *Mundinia* clade, confirming their taxonomic placement, while those of *L. amazonensis* and *L. major* group within the *Leishmania* clade. *Crithidia mellificae* was used as the outgroup. Color gradients correspond to the posterior probability (BI, left) and bootstrap support (ML, right) scales

The BI and ML phylogenetic analyses based on ITS1 sequences yielded congruent tree topologies, consistently grouping *Leishmania* species into four major, well-supported clades corresponding to the subgenera *Leishmania*, *Sauroleishmania*, *Viannia*, and *Mundinia* (Fig. 7). Each clade was strongly supported by high posterior probabilities and bootstrap values, demonstrating the robustness of the phylogenetic relationships inferred from the ITS1 marker.

The *Mundinia* clade was distinctly separated from other subgenera and included both *L. martiniquensis* and *L. orientalis*. Most sequences derived from *Forcipomyia* specimens in this study clustered within the *Mundinia* clade with strong nodal support, forming two discrete subclades: one containing *L. orientalis* (NST_1, NST_12, NST_28, and NST_44) and the other comprising *L. martiniquensis* (NST_1, NST_28, NST_38, NST_44, and NST_102). These sequences grouped monophyletically with known Thai isolates of *L. martiniquensis* and *L. orientalis*, confirming their taxonomic placement within *Mundinia*. The ITS1 sequences detected in this study are also genetically identical to those previously reported from human cases of *L. orientalis* and *L. martiniquensis* in Southern Thailand.

Clades representing the *Viannia* and *Leishmania* subgenera were also clearly delineated. Notably, *L. major* (NST_1) and *L. amazonensis* (NST_12 and NST_28), both members of the *Leishmania* subgenus, were molecularly detected in Thai midge specimens for the first time. This finding expands the known geographic distribution of these species. It suggests that both autochthonous and introduced *Leishmania* lineages coexist in Thailand, potentially involving novel parasite-midge associations within the *Lasiohelea* complex.

### Vertebrate host identification

Vertebrate *cox*1 amplicons were successfully amplified from the blood-engorged specimen (NST_14) and sequenced using MinION^®^ amplicon sequencing. After quality filtering, 33,973 high-quality reads were analyzed using amplicon_sorter.py to generate consensus sequences. A single consensus sequence was produced and trimmed to remove forward and reverse primer regions containing degenerate positions that could affect similarity scores when compared with GenBank references. The trimmed consensus sequence matched the mitochondrial *cox*1 reference sequence of the red junglefowl (*Gallus gallus spadiceus*) (GenBank: GU261706) with 100% identity. This finding is consistent with field observations at the collection site, where free-ranging red junglefowl were reported in nearby households, suggesting that these birds were a likely blood meal source for the engorged *Forcipomyia* specimen.

## Discussion

Morphological descriptions of *Forcipomyia* (*Lasiohelea*) species in Asia remain limited and often incomplete, with female stages frequently remaining undescribed. This limited availability of diagnostic information complicates species-level identification, as key features such as mandibular dentition, cibarial armature, and spermathecal morphology are subtle and require specialist interpretation. To overcome these limitations, we focused on female specimens possessing a single spermatheca, a key diagnostic feature of *Lasiohelea* females, and applied an integrative framework combining detailed morphological examination with DNA barcoding based on *cox*1 reference sequences. This approach, supported by phylogenetic reconstruction and multiple species delimitation analyses, improved species-level resolution and reduced reliance on morphology alone.

*Cox*1-based phylogenetic analyses robustly resolved major *Forcipomyia* subgenera, including *Panhelea*, *Euprojoannisia*, *Lasiohelea*, *Forcipomyia*, and *Caloforcipomyia*, with strong statistical support. Within *Lasiohelea*, four well-supported clusters corresponded to morphologically distinct lineages closely associated with *F.* (*L.*) *peditata*, *F.* (*L.*) *humilavolita*, *F.* (*L.*) *taiwana*, and *F.* (*L.*) *parvitas*. The latter was consistently recovered as a distinct species across BLASTn identification, phylogenetic inference, and species delimitation analyses, providing strong molecular confirmation that the specimens examined in this study belong to *F.* (*L.*) *parvitas*. In contrast, several specimens related to *F.* (*L.*) *peditata* and *F.* (*L.*) *humilavolita* exhibited relatively low sequence similarity to available reference sequences despite displaying highly consistent or nearly identical morphological traits. Multiple species delimitation methods concordantly recognized these lineages as putatively distinct species, indicating that substantial genetic divergence may occur despite limited morphological differentiation and suggesting the presence of cryptic diversity shaped by broad geographic distribution and regional population divergence [14, 46, 47].

Unassigned Ceratopogonidae lineages (groups 1 and 2), together with *F.* (*Euprojoannisia*) *fuscimana*, further suggest the presence of previously unrecorded or potentially locally structured taxa, highlighting the underestimated diversity of ceratopogonids in Thailand. The phylogenetic clustering of Thai *Forcipomyia* (*Lasiohelea*) sequences with those from India and China indicates a broad Asian distribution and close genetic relationships with *F. peditata* (India) [39], *F. humilavolita* (China) [48], *F. taiwana* (China), and *F. parvitas* (India) [39], suggesting historical geographic connectivity and potential gene flow across regional populations [24].

Morphological differentiation in mandibular dentition, maxillary palps, and cibarial armature across *Forcipomyia* subgenera supports ecological and functional divergence, consistent with feeding specialization reported in other Ceratopogonidae [49–52]. In particular, *Lasiohelea* species exhibit more pronounced mandibular dentition, well-developed sensory pits, and distinct cibarial armature compared with non-*Lasiohelea* lineages, indicating adaptive divergence associated with foraging strategies [24, 26, 39–42]. Collectively, our findings highlight the importance of integrating morphological and molecular approaches for resolving taxonomic complexity in *Forcipomyia* (*Lasiohelea*). This integrative framework not only improves species-level resolution but also reveals divergent genetic lineages and hidden taxonomic diversity that would otherwise remain undetected using morphology alone.

A key finding of this study was the molecular detection of multiple *Leishmania* species, including *L. martiniquensis*, *L. orientalis*, *L. amazonensis*, and *L. major*, in six of seven tested *Forcipomyia* (*Lasiohelea*) specimens. Notably, ITS1 sequences generated in this study were genetically identical to those previously reported from human cases of *L. martiniquensis* and *L. orientalis* in endemic areas of Thailand, supporting a close epidemiological association between parasite populations detected in *Forcipomyia* (*Lasiohelea*) midges and those infecting humans [11–13]. The predominance of *L. martiniquensis* and *L. orientalis*, both members of the subgenus *Mundinia*, is consistent with the known endemic *Leishmania* fauna of Thailand and suggests the continued circulation of these parasites in endemic areas of Thailand [5–8, 10–16].

The detection of *L. amazonensis* and *L. major* in Thai *Forcipomyia* (*Lasiohelea*) specimens represents a novel finding, as neither species has been reported as an autochthonous agent of leishmaniasis in Thailand. *Leishmania amazonensis* is mainly distributed in the New World [53, 54], whereas *L. major* is typically associated with transmission foci in Africa, the Middle East, and Central Asia [55–57]. Previous reports have documented several non-autochthonous *Leishmania* species, including the *L. donovani/L. infantum* complex, *L. lainsoni*, and *L. major*, in HIV/AIDS patients from Southern Thailand [30]. The occurrence of these parasites in midges may reflect exposure to infected humans or animals introduced from outside endemic transmission regions, providing opportunities for parasite acquisition during blood feeding [30, 58]. The coexistence of *Leishmania* species with diverse epidemiological origins within the same geographic area suggests a complex ecological setting in which humans, domestic animals, and hematophagous insects interact, potentially facilitating the maintenance and circulation of multiple parasite species [11–13, 59].

Although *Culicoides* species are recognized as important ceratopogonid vectors of vertebrate pathogens [2], evidence supporting a role for *Forcipomyia* (*Lasiohelea*) in pathogen transmission remains limited. Previous studies have implicated members of the *Lasiohelea* subgenus in the transmission of *L.* (*M.*) *macropodum,* the causative agent of cutaneous leishmaniasis in red kangaroos [20, 21]. In the present study, *Leishmania* DNA was detected in six of seven specimens examined, including mixed-species detections, demonstrating natural exposure of *Forcipomyia* (*Lasiohelea*) midges to *Leishmania* parasites in endemic environments. These findings support further investigation into their potential involvement in local transmission cycles.

According to the Killick-Kendrick criteria, a putative vector should demonstrate four key characteristics: (1) feeding behavior that includes humans and, for zoonotic pathogens, the appropriate reservoir hosts; (2) the capacity to support parasite development after the blood meal has been digested; (3) carriage of parasite strains that are indistinguishable from those infecting vertebrate hosts; and (4) the ability to transmit the parasite during blood feeding [60]. The present study provides evidence for the third criterion by demonstrating that *Leishmania* species detected in *Forcipomyia* (*Lasiohelea*) specimens are genetically indistinguishable from parasites previously reported in human infections from endemic areas of Thailand. However, molecular detection of parasite DNA alone is insufficient to confirm vector competence. For successful transmission, *Leishmania* parasites must survive digestion of the blood meal, traverse the peritrophic matrix, adhere to the midgut epithelium, and develop into infective metacyclic promastigotes [17–19, 61, 62]. Stronger evidence would require demonstration of persistent natural infections beyond residual blood meals or the observation of infective parasite stages within field-collected midges [63]. Conclusive proof of vector competence requires maintaining midges under laboratory conditions and conducting controlled experimental infections and transmission trials, or at least dissections of wild-caught midges that reveal metacyclic promastigotes in the stomodeal valve, thereby satisfying the second and fourth Killick-Kendrick criteria [17–19, 64].

In contrast to the molecular evidence supporting the third Killick-Kendrick criterion, evaluation of the first criterion requires information on host-feeding behavior [60]. Species within the subgenus *Lasiohelea* exhibit diverse host preferences. As previously recorded, *Forcipomyia* (*Lasiohelea*) *sibirica* feeds on humans and other endothermic vertebrates, while *F.* (*L.*) *velox* targets amphibians and transmits the filarial nematode *Icosiella neglecta* to frogs of the *Rana esculenta* complex [65]. *Forcipomyia* (*L.*) *taiwana* displays strong anthropophily, frequently causing bites, nuisances, and allergic reactions in Taiwan and Southern China [24, 41, 66–68]. Similarly, *Forcipomyia* (*Lasiohelea*) *townsvillensis* bites humans, pets, and livestock, with evidence implicating it in the transmission of arboviruses and the cattle nematode *Onchocerca gibsoni* [69]. In addition, *Forcipomyia* (*Lasiohelea*) midges are anautogenous and require blood meals for egg development, increasing opportunities for repeated host contact and pathogen acquisition throughout their life cycle [70].

Most *Forcipomyia* (*Lasiohelea*) species are also active during daylight hours, overlapping with periods of intense human activity and potentially increasing opportunities for human exposure to biting midges [71]. Although vertebrate *cox*1 metabarcoding identified red junglefowl as the blood source of the single engorged specimen examined in this study, this observation does not satisfy the first Killick-Kendrick criterion, which requires evidence of human blood feeding [60]. Nevertheless, the finding suggests that *Forcipomyia* (*Lasiohelea*) midges occur in peridomestic environments where humans and animals coexist, potentially facilitating interactions between midges, hosts, and parasite reservoirs. However, since only one blood-fed specimen was analyzed, firm conclusions regarding local host preferences cannot be drawn. More extensive sampling is needed to better understand the host range and feeding behavior in this region.

Overall, this study provides the first integrated molecular and morphological characterization of *Forcipomyia* (*Lasiohelea*) midges from Thailand and the first molecular evidence of multiple *Leishmania* species associated with wild-caught *Forcipomyia* (*Lasiohelea*) midges in a leishmaniasis-endemic area. The integration of DNA barcoding with traditional morphological analysis enabled reliable species identification and revealed previously unrecognized genetic diversity within the *Lasiohelea* complex. Together with the detection of both autochthonous and non-autochthonous *Leishmania* species, these findings enhance current understanding of the ecological associations between ceratopogonid midges and *Leishmania* parasites. Further studies focusing on vector competence, parasite localization, and host-feeding behavior are needed to clarify the epidemiological significance of *Forcipomyia* (*Lasiohelea*) midges in *Leishmania* transmission. Such investigations will contribute to a more comprehensive understanding of leishmaniasis ecology and may help reassess the role of ceratopogonid midges in transmission cycles across Southeast Asia.

## Conclusions

This study demonstrates the value of integrating molecular approaches with traditional morphological methods to assess the diversity of *Forcipomyia* (*Lasiohelea*) midges and detect *Leishmania* parasites in *Lasiohelea* specimens from a leishmaniasis focus in Southern Thailand. Molecular analyses identified several putatively distinct species closely related to recognized *Lasiohelea* taxa, highlighting the need for robust taxonomic frameworks and complementary morphological and molecular evidence to accurately delimit species and characterize the diversity of these biting midges. The detection of multiple *Leishmania* species in *Lasiohelea* specimens suggests a complex parasite-midge association and indicates the co-circulation of different parasites within the local environment. Although the detection of *Leishmania* DNA does not directly demonstrate vector competence, it supports the possibility that *Forcipomyia* (*Lasiohelea*) midges are involved in local parasite transmission cycles. Future studies focusing on vector competence assays, parasite development within midges, and detailed host-vector interactions will be essential to clarify their precise role in *Leishmania* transmission. These findings support enhanced surveillance and targeted research on ceratopogonid midges in Southeast Asia, recognizing their potential epidemiological relevance in leishmaniasis transmission dynamics. Such efforts will contribute to improved assessment of *Leishmania* transmission risk associated with ceratopogonid midges and facilitate the development of more effective control strategies in the region.

## Supplementary Information

Additional file 1.Table S1 Molecular identification of from Nakhon Si Thammarat Province, Southern Thailand, based on BOLD and BLASTn analyses.

## Data Availability

All data supporting the results of this study are available in the paper and its supplementary information.
