# Supplementary material for: Integrative morphological and DNA barcoding analysis of Forcipomyia (Lasiohelea) midges: cryptic diversity and first molecular detection of multiple Leishmania species in a leishmaniasis-endemic focus in Southern Thailand
Source: Parasit Vectors. 2026 Jun 30;19:396. doi: 10.1186/s13071-026-07544-5 (PMC13576339; doi:10.1186/s13071-026-07544-5)
Supplement: Supplementary file 1 [file 13071_2026_7544_MOESM1_ESM.docx]

**Table S1** Molecular identification of Forcipomyia spp. from Nakhon Si Thammarat Province, Southern Thailand, based on BOLD and BLASTn analyses

| **Sample ID** | **BOLD Search** | | **BLASTn Search** | |
| --- | --- | --- | --- | --- |
|  | **Closest Species (BOLD Process ID)** | **Identity**  **(%)** | **Closest species (GenBank accession number)** | **Identity**  **(%)** |
| NST_2 | Ceratopogonidae sp. (GMMBD354-16) | 100 | Ceratopogonidae sp. (KM959488) | 86.10 |
| NST_8 | Ceratopogonidae sp. (GMMBD354-16) | 100 | Ceratopogonidae sp. (KM959488) | 86.42 |
| NST_11 | Ceratopogonidae sp. (GMMBD354-16) | 100 | Ceratopogonidae sp. (KM959488) | 86.42 |
| NST_19 | Ceratopogonidae sp. (GMMNC205-18) | 99.85 | Ceratopogonidae sp. (KM959488) | 86.57 |
| NST_26 | Ceratopogonidae sp. (GMMBD354-16) | 100 | Ceratopogonidae sp. (KM959488) | 86.23 |
| NST_47 | Ceratopogonidae sp. (GMMBD354-16) | 100 | Ceratopogonidae sp. (KM959488) | 86.18 |
| NST_58 | Ceratopogonidae sp. (GMMBD354-16) | 100 | Ceratopogonidae sp. (KM959488) | 86.42 |
| NST_65 | Ceratopogonidae sp. (GMMBD354-16) | 100 | Ceratopogonidae sp. (KM959488) | 86.42 |
| NST_81 | Ceratopogonidae sp. (GMMBD354-16) | 100 | Ceratopogonidae sp. (KM959488) | 86.13 |
| NST_89 | Ceratopogonidae sp. (GMMBD354-16) | 100 | Ceratopogonidae sp. (KM959488) | 86.42 |
| NST_108 | Ceratopogonidae sp. (GMMBD354-16) | 100 | Ceratopogonidae sp. (KM959488) | 86.42 |
| NST_130 | Ceratopogonidae sp. (GMMBD354-16) | 100 | Ceratopogonidae sp. (KM959488) | 86.42 |
| NST_145 | Ceratopogonidae sp. (GMMBD354-16) | 100 | Ceratopogonidae sp. (KM959488) | 86.42 |
| NST_150 | Ceratopogonidae sp. (GMMBD354-16) | 100 | Ceratopogonidae sp. (KM959488) | 86.42 |
| NST_161 | Ceratopogonidae sp. (GMMBD354-16) | 100 | Ceratopogonidae sp. (KM959488) | 86.05 |
| NST_165 | Ceratopogonidae sp. (GMMBD354-16) | 100 | Ceratopogonidae sp. (KM959488) | 86.42 |
| NST_170 | Ceratopogonidae sp. (GMMNC205-18) | 99.85 | Ceratopogonidae sp. (KM959488) | 86.27 |
| NST_173 | Ceratopogonidae sp. (GMMNC205-18) | 99.85 | Ceratopogonidae sp. (KM959488) | 86.27 |
| NST_175 | Ceratopogonidae sp. (GMMBD354-16) | 100 | Ceratopogonidae sp. (KM959488) | 86.42 |
| NST_191 | Ceratopogonidae sp. (GMMBD354-16) | 100 | Ceratopogonidae sp. (KM959488) | 86.42 |
| NST_193 | Ceratopogonidae sp. (GMMBD354-16) | 100 | Ceratopogonidae sp. (KM959488) | 86.42 |
| NST_221 | Ceratopogonidae sp. (GMMBD354-16) | 100 | Ceratopogonidae sp. (KM959488) | 86.42 |
| NST_228 | Ceratopogonidae sp. (GMMNC205-18) | 99.85 | Ceratopogonidae sp. (KR637506) | 86.47 |
| NST_241 | Ceratopogonidae sp. (GMMBD354-16) | 100 | Ceratopogonidae sp. (KM959488) | 86.44 |
| NST_3 | *Forcipomyia* sp. (GMMSI498-18) | 99.85 | Ceratopogonidae sp. (KX051897) | 97.23 |
| NST_4 | *Forcipomyia* sp. (GMMNM199-18) | 99.85 | Ceratopogonidae sp. (KX051897) | 97.54 |
| NST_5 | *Forcipomyia* sp. (GMMSJ1041-18) | 99.85 | Ceratopogonidae sp. (KX051897) | 97.23 |
| NST_9 | *Forcipomyia* sp. (GMMNA709-18) | 100 | Ceratopogonidae sp. (KX051897) | 97.23 |
| NST_10 | *Forcipomyia* sp. (GMPXA4703-23) | 100 | Ceratopogonidae sp. (KX051897) | 98.30 |
| NST_16 | *Forcipomyia* sp. (GMMNA709-18) | 100 | Ceratopogonidae sp. (KX051897) | 97.22 |
| NST_22 | *Forcipomyia* sp. (GMTTB15771-23) | 99.69 | Ceratopogonidae sp. (KX051897) | 97.69 |
| NST_29 | *Forcipomyia* sp. (GMTTA300-18) | 99.53 | Ceratopogonidae sp. (KX051897) | 96.76 |
| NST_32 | *Forcipomyia* sp. (GMMNL869-18) | 99.69 | Ceratopogonidae sp. (KX051897) | 97.38 |
| NST_34 | *Forcipomyia* sp. (GMTTB15771-23) | 100 | Ceratopogonidae sp. (KX051897) | 97.69 |
| NST_36 | *Forcipomyia* sp. (GMMSG558-18) | 100 | Ceratopogonidae sp. (KX051897) | 97.84 |
| NST_40 | *Forcipomyia* sp. (GMMSF302-18) | 99.85 | Ceratopogonidae sp. (KX051897) | 98.15 |
| NST_45 | *Forcipomyia* sp. (GMMNA709-18) | 100 | Ceratopogonidae sp. (KX051897) | 97.22 |
| NST_52 | *Forcipomyia* sp. (GMMNM340-18) | 99.85 | Ceratopogonidae sp. (KX051897) | 97.99 |
| NST_57 | *Forcipomyia* sp. (GMTKG383-18) | 100 | Ceratopogonidae sp. (KX051897) | 97.23 |
| NST_71 | *Forcipomyia* sp. (GMMGT3012-18) | 99.85 | Ceratopogonidae sp. (KX051897) | 97.07 |
| NST_79 | *Forcipomyia* sp. (GMPMA3565-18) | 99.85 | Ceratopogonidae sp. (KX051897) | 97.84 |
| NST_95 | *Forcipomyia* sp. (GMTKV254-18) | 99.85 | Ceratopogonidae sp. (KX051897) | 97.53 |
| NST_97 | *Forcipomyia* sp. (GMMNA709-18) | 100 | Ceratopogonidae sp. (KX051897) | 96.92 |
| NST_138 | *Forcipomyia* sp. (GMTTB5675-23) | 99.54 | Ceratopogonidae sp. (KX051897) | 97.22 |
| NST_143 | *Forcipomyia* sp. (GMMSJ1041-18) | 98.60 | Ceratopogonidae sp. (KX051897) | 96.15 |
| NST_153 | *Forcipomyia* sp. (GMMNA709-18) | 100 | Ceratopogonidae sp. (KX051897) | 97.07 |
| NST_155 | *Forcipomyia* sp. (GMPXA4703-23) | 100 | Ceratopogonidae sp. (KX051897) | 98.15 |
| NST_168 | *Forcipomyia* sp. (GMPMA427-18) | 100 | Ceratopogonidae sp. (KX051897) | 98.15 |
| NST_169 | *Forcipomyia* sp. (GMTTB6407-23) | 98.93 | Ceratopogonidae sp. (KX051897) | 96.60 |
| NST_181 | *Forcipomyia* sp. (GMMSI1647-18) | 99.85 | Ceratopogonidae sp. (KX051897) | 97.69 |
| NST_183 | *Forcipomyia* sp. (GMTTA300-18) | 100 | Ceratopogonidae sp. (KX051897) | 97.53 |
| NST_185 | *Forcipomyia* sp. (GMMNA709-18) | 100 | Ceratopogonidae sp. (KX051897) | 97.22 |
| NST_187 | *Forcipomyia* sp. (GMMSJ1041-18) | 99.84 | Ceratopogonidae sp. (KX051897) | 97.22 |
| NST_198 | *Forcipomyia* sp. (GMMSM670-18) | 99.69 | Ceratopogonidae sp. (KX051897) | 97.69 |
| NST_203 | *Forcipomyia* sp. (GMPXA4703-23) | 100 | Ceratopogonidae sp. (KX051897) | 98.30 |
| NST_205 | *Forcipomyia* sp. (GMMSI1647-18) | 100 | Ceratopogonidae sp. (KX051897) | 97.84 |
| NST_209 | *Forcipomyia* sp. (GMMSI1647-18) | 99.85 | Ceratopogonidae sp. (KX051897) | 97.69 |
| NST_211 | *Forcipomyia* sp. (GMMGT3012-18) | 99.85 | Ceratopogonidae sp. (KX051897) | 97.07 |
| NST_213 | *Forcipomyia* sp. (GMPMF906-18) | 99.85 | Ceratopogonidae sp. (KX051897) | 97.84 |
| NST_215 | *Forcipomyia* sp. (GMMNA709-18) | 99.85 | Ceratopogonidae sp. (KX051897) | 97.07 |
| NST_233 | *Forcipomyia* sp. (GMMSJ975-18) | 99.85 | Ceratopogonidae sp. (KX051897) | 97.69 |
| NST_245 | *Forcipomyia* sp. (GMMNM229-18) | 99.85 | Ceratopogonidae sp. (KX051897) | 97.22 |
| **NST_82** | ***Forcipomyia* sp. (GBAAX68727-24)** | **98.46** | ***Forcipomyia fuscimana* (MZ539943)** | **98.46** |
| **NST_103** | ***Forcipomyia* sp. (GBAAX68727-24)** | **98.29** | ***Forcipomyia fuscimana* (MZ539943)** | **98.29** |
| **NST_111** | ***Forcipomyia* sp. (TIGMV19826-25)** | **98.33** | ***Forcipomyia fuscimana* (MZ539943)** | **98.29** |
| **NST_1** | ***Forcipomyia sp.* (GBAAX14159-24)** | **91.31** | ***Forcipomyia peditata* (OR117382)** | **91.31** |
| **NST_14** | ***Forcipomyia* sp. (GBAAX14159-24)** | **91.31** | ***Forcipomyia peditata* (OR117382)** | **91.31** |
| **NST_28** | ***Forcipomyia* sp. (GBAAW80933-24)** | **98.32** | ***Forcipomyia parvitas* (OM632702)** | **98.32** |
| **NST_102** | ***Forcipomyia* sp. (GBAAW80933-24)** | **98.88** | ***Forcipomyia parvitas* (OM632702)** | **98.88** |
| **NST_12** | **Ceratopogonidae sp. (GMMSE1124-18)** | **99.69** | ***Forcipomyia taiwana* (KF528690)** | **88.82** |
| **NST_38** | **Ceratopogonidae sp. (GMMSM046-18)** | **99.70** | ***Forcipomyia humilavolita* (PP199400)** | **87.98** |
| **NST_44** | **Ceratopogonidae sp. (GMMSU20880-25)** | **100** | ***Forcipomyia humilavolita* (PP199400)** | **88.12** |
